# Comparative Study of Chemical, Biochemical Characteristic and ATR-FTIR Analysis of Seeds, Oil and Flour of the Edible Fedora Cultivar Hemp (*Cannabis sativa* L.)

**DOI:** 10.3390/molecules24010083

**Published:** 2018-12-27

**Authors:** Francesco Siano, Stefania Moccia, Gianluca Picariello, Gian Luigi Russo, Giuseppe Sorrentino, Michele Di Stasio, Francesco La Cara, Maria Grazia Volpe

**Affiliations:** 1 Istituto di Scienze dell’Alimentazione, Consiglio Nazionale delle Ricerche (CNR), Via Roma 64, I-83100 Avellino, Italy; francesco.siano@isa.cnr.it (F.S.); picariello@isa.cnr.it (G.P.); glrusso@isa.cnr.it (G.L.R.); michele.distasio@isa.cnr.it (M.D.S.); 2 Istituto di Biologia Agro-ambientale e Forestale, Consiglio Nazionale delle Ricerche (CNR), Via Pietro Castellino 111, I-80131 Napoli, Italy; stefania.moccia@cnr.it (S.M.); francesco.lacara@cnr.it (F.L.C.); 3 Istituto per i Sistemi Agricoli e Forestali del Mediterraneo, Consiglio Nazionale delle Ricerche (CNR), Via Cupa Patacca 85, I-80056 Ercolano (NA), Italy; giuseppe.sorrentino@cnr.it

**Keywords:** *Cannabis sativa* L., polyphenols, antioxidants, lipidic profile, minerals, ATR-FTIR

## Abstract

A series of chemical and biochemical parameters of edible hemp resources (seeds, oil, and flour) from the monoecious EU registered hemp genotype Fedora, was determined, including fatty acid profile, phytosterol composition, total phenolics, antioxidant activity, macro- and micro-elements. The fatty acid ω-3/ω-6 approached the nutritionally optimal 3/1 ratio. β-sitosterol and other phytosterols sterols dominated the unsaponifiable fraction. Hemp seeds, flour, and oil contained 767 ± 41, 744 ± 29, and 21 ± 5 mg GAE kg^−1^ total polyphenols, respectively. The antioxidant potential of Fedora flour and seeds, evaluated through the DPPH (2,2-Diphenyl-1-picrylhydrazyl) assay, was higher than that of oil. K and Mg were the most abundant macro-elements, particularly in flour, while the concentration of trace elements was Fe > Cu > Ni > Mn. The presence of an array of bioactive compound candidate Fedora products as health-promoting food matrices. The ATR-FTIR spectra of hemp-derived products indicated the proximate composition of macro-nutrients.

## 1. Introduction

*Cannabis sativa* plant, known as hemp, is one of the oldest domesticated crops. Hemp has been cultivated for years to produce paper, textiles, and cordage. When utilized as a food source, hemp seeds are processed by cold pressing, resulting in oil and pressed cakes (flour), with alleged excellent nutraceutical properties [1]. Hemp oil is rich in nutrients and health-promoting components, including vitamins, mineral salts, amino acids, and phytosterols, etc. In this sense, the most interesting property is the relatively high content of α-linolenic essential fatty acid, belonging to the ω-3 series. In general, hemp seed oil is high in polyunsaturated fatty acids (PUFAs), especially linoleic (18:2, ω-6) and α-linolenic (18:3, ω-3) essential fatty acids [2]. The ω-6 to ω-3 ratio (ω-6/ω-3) approaches 3:1 which is considered desirable to reduce the risk of dyslipidaemia-associated diseases [3,4].

Hemp health benefits are also associated with phenolic compounds [5], which occur in variable amounts in almost all classes of plant-derived foods and agro-industrial residues. Epidemiological, clinical, and nutritional studies strongly support the evidence that dietary phenolic compounds are effective in the prevention of common diseases, including cancer, neurodegenerative diseases, gastrointestinal disorders, and others [6,7]. A large number of polyphenols has been identified in hemp, especially flavonoids such as flavanones, flavanols, flavonols, and isoflavones [8]. Hemp flavones and flavonols exert a wide range of biological effects, such as anti-inflammatory, anti-cancer, and neuro-protective properties [9]. In addition, apigenin has been shown to play anxiolytic and estrogenic roles [10,11]. Several simple and complex lignanamides, also endowed with uncommon and varying bioactive properties, are among the most abundant (poly-) phenolics of hemp seeds and cake flour [12]. 

Hemp seeds also supply a range of macro- and micro-elements to humans. The deficiency of mineral nutrients, such as Fe, I, and Zn, is a growing nutritional problem in human populations, while uptake of other elements, such as Ca, K, Mg, and Se, can be poor in the diets of some specific populations.

Although *C. sativa* contains only trace amounts of tetrahydrocannabinols (THCs), the psychotropic components from the congener *C. indica*, its growing has been long forbidden in Western Countries for the precautionary purpose [1]. However, in the last decades a renewed interest in the utilization of this crop with low-THC (0–0.2%), for non-drug purposes, and as a raw material to produce gluten-free foods, fuels, textiles, paper or even biodegradable plastics, took place [13]. The versatility of hemp seeds may lead to the sustainable development of numerous products to be employed in the food, cosmetic, therapeutic and nutraceutical industries [14]. Nowadays, cold pressed hemp seed oils, flour, and seeds are indeed commercially available. 

In spite of the renovated interest for this crop, biochemical data about hemp products and by-products are still fragmentary. In particular, it appears missing a comprehensive record cataloguing the qualitative and quantitative properties of the edible resources from individual cultivars.

This study reports on the physico-chemical, chemical, and biochemical characterization of hemp seeds, oil, and flour (*Cannabis sativa* L.) from the monoecious genotype *Fedora*, which is among the most popular hemp variety and it is listed within the EU database of admitted agricultural species [15]. The main aim of the research was to monitor the distribution of hemp seed components in the flour or in the oil, after fractionation by cold-pressing. In particular, both the saponificable and un-saponificable lipid fraction, accounting for 25–35% by weight of hemp seed, and the macro- and micro-elements, as well as the total polyphenols content and the antioxidant activity (DPPH method), have been determined. Finally, possible cultivar characteristic traits to be monitored for traceability were assessed using ATR-FTIR spectroscopy. 

## 2. Materials and Methods

### 2.1. Materials 

The edible hemp resources, hemp seeds, hemp flour cake, and cold-pressed hemp seed oil, obtained from the *Fedora* cultivar, were provided by “Lentamente Società Cooperativa, Agricola” (Torrecuso BN, Italy). Seeds were collected during three consecutive years and refrigerated at −20 °C soon after harvesting. Hemp products were obtained from seeds mixed in equal proportions among the three collections, in order to average the composition over different harvests. 

Before analysis, seeds were ground by a hand-held electric coffee mill. Samples were immediately processed. Chemicals were from Sigma-Aldrich (St. Louis, MI, USA) and solvents, of the highest purity degree available, were from Carlo Erba (Milan, Italy). 

### 2.2. Proximate Composition

Seed and flour proximate composition (moisture, crude protein, crude fat, total ash) was determined using the Official Methods of Analysis of AOAC International [16]. Total carbohydrates were determined by difference in percentages: 100% − (crude protein + crude fat + total ash + moisture)%. Lipids were extracted from seeds and flour with the Soxhlet method and stored at −20 °C until further analysis.

### 2.3. Polyphenols Extraction

Polyphenols extraction has been carried out according to Vonapartis et al. [17] with some modifications. Briefly, 1 g of sample was suspended in 10 mL of aqueous methanol 80% (*v/v*). The mixture was vortexed, sonicated in the dark, at 4 °C, for 30 min. Thereafter the mixture was centrifuged at 3500× *g* at 4 °C for 10 min. The supernatant was collected and the pellet was extracted again with 6 mL of methanol 80% (*v/v*) and centrifuged. The supernatants were pooled and kept at −20 °C until analysis. 

### 2.4. Total Polyphenols

Total polyphenols were measured with the Folin-Ciocalteu method [18], using gallic acid as the standard. Total phenols were expressed as mg of gallic acid equivalents (GAE) per kg of sample.

Alternatively, total polyphenols were determined by reversed phase-high performance liquid chromatography (RP-HPLC), adapting the International Olive Council (IOC) method for the determination of the olive oil biophenols [19]. 

To this purpose, 2 mL aliquots of the polyphenol extracts were dried in a Savant speed-vac, re-suspended in 0.1% trifluoroacetic acid (TFA). One-tenth of the polyphenol extracts from seeds and flour, and half of the oil extract were separated by RP-HPLC using a modular HP 1100 chromatographer (Agilent, Palo Alto, CA, USA) equipped with a diode array detector (DAD). The stationary phase was a C_18_ reversed-phase column 250 × 2.1 mm i.d., 4 μm particle diameter (Jupiter Phenomenex, Torrance, CA, USA), at 37 °C with a thermostatic oven. Separations were carried out at a 0.2 mL/min constant flow rate, applying a 5–65% gradient of the organic modifier (solvent B: Acetonitrile/0.1% TFA) in 5–65 min, following 5 min of isocratic elution at 5% B. At 66 min the % B increased up to 100%. Solvent A was 0.1% TFA in HPLC-grade water. Separations were monitored at λ = 450, 360, 320, and 280 nm wavelengths, and peaks were integrated using the HPLC ChemStation software vers. A.07.01 (Agilent). Samples were run in triplicate and the peak area was averaged among the technical replicates. Practically, polyphenols were separated, determined at 280 nm and quantified using caffeic acid as the internal standard, considering the same response factor for all of the phenolic compounds [20]. HPLC-determined polyphenols were expressed as caffeic acid equivalents (CAE) for Kg of the sample.

### 2.5. DPPH Radical Scavenging Activity 

One millilitre of freshly prepared DPPH radical solution (0.125 mM) was added to 1 mL of the extract (0.2 g mL^−1^) prepared as described and mixed well to start the radical-antioxidant reaction. The absorbance at 517 nm was determined against a blank of pure methanol after 0, 2, 4, 6, 8, 10, 15, and 20 min of reaction, and used to estimate the remaining radical levels according to the standard curve. The percent inhibition was calculated, after 15 min of reaction, according to Lee et al. [21] using the following equation: % inhibition = [(absorbance of control – absorbance of the test sample)/absorbance of control] × 100. A calibration curve for DPPH inhibition was obtained with Trolox^®^ (Sigma) assayed in the 0.1–10 mM concentration range. 

### 2.6. Preparation of FAME 

Analysis of the FAME (Fatty Acid Methyl Ester) was performed according to Siano et al. [22]. The samples of crude extracted oil and Soxhelet-extracted lipids from seeds and flour (about 0.2 g) were transferred into Pyrex test tubes with screw caps, and 2 mL of 1.25 N HCl-CH_3_OH solution was added. Samples were incubated in a water bath at 90 °C for 60 min. FAME was extracted with *n*-hexane, after the addition of 2 mL of distilled water. The organic phase was filtered using Millex 0.45 μm PVDF disposable syringe filters (EMD Millipore Corp., Billerica, MA, USA) and 1 μL was directly injected into the gas chromatograph for analysis.

### 2.7. GC-FID Analysis of Fatty Acids 

FAME, prepared as reported above, were analysed with a Trace GC gas chromatographer (Thermo Scientific, Inc., San Jose, CA, USA) equipped with an FID (Flame Ionization Detector), using an SP-2560 (100 m × 0.25 mm × 0.20 μm) capillary column (Supelco, Sigma-Aldrich). Samples were introduced through a split-splitless injection system of an AS 3000 autosampler in split mode (ratio 1:100) at 260 °C. The oven temperature program started at 140 °C (held for 5 min) and linearly increased to 260 °C (4 °C min^−1^) up to the end of the analysis, according to previously described operating conditions (Siano, et al., 2016). FID temperature was 260 °C. The fatty acid composition of all samples was obtained by comparison with the retention times of the standard mixture FAME 37 components (Sigma-Aldrich) and was expressed as a percentage area. 

### 2.8. GC-FID Analysis of Unsaponificable

All oil samples (crude extracted oil, Soxhelet-extracted lipids from seed and flour) were saponified according to the method of Caligiani et al. [23]. The unsaponified components were extracted in diethyl ether and washed with water until neutral reaction occured. The ether phase was dehydrated with anhydrous sodium sulfate, filtered on paper and finally dried under vacuum. The residue oil was dissolved in 50 mL of *n*-hexane and analyzed using the above chromatographer equipped with an RTX-5, 30 m × 0.25 mm × 0.25 μm column (Restek, Bellefonte, PA, USA). Samples (1 μL) were introduced through the autosampler in 1:10 split mode at 250 °C. The oven temperature program started at 200 °C (held for 2 min) and linearly increased to 300 °C (20 °C min^−1^) at the end of the analysis. Plant sterol mix (Matreya, State College, PA, USA), was used as external standards for qualitative and quantitative determinations. Data were recorded and processed using the ChromQuest 5.0 software (Thermo).

### 2.9. Mineralization of Samples

Acid digestion of bean samples was performed according to Volpe et al. [24]. To one gram of dried sample, 16 mL of HNO_3_/H_2_O_2_ (6/2, *v/v*) of solution were added. The mixture was heated to 130 °C until the solution became transparent. After cooling, the solution was filtered and diluted to 25 mL in a volumetric flask. The results were analyzed with Inductively Coupled Argon Plasma Optical Emission Spectrometers (ICP-OES).

### 2.10. ICP-OES Analysis

The elemental analysis of macro and trace elements were measured by ICP-OES with iCAP 7000 Series (Thermo Scientific), equipped with ASX-520 autosampler (CETAC™). A calibration curve was constructed using three standard solutions for each element. In order to prevent interference to calibration solutions, 100 µg L^−1^ yttrium solution (Trace Cert, Fluka) was used as internal standard. The elements contents were calculated by using standard curves and the final concentrations were expressed as g element kg^−1^ dry weight for macro-elements, while they were expressed as mg element kg^−1^ dry weight for micro-elements. 

### 2.11. ATR-FTIR Analysis

Attenuated Total Reflectance-Fourier Transform Infrared (ATR-FTIR) analyses were performed using a Spectrum 400 spectrophotometer (Perkin Elmer, Waltham, MA, USA), equipped with a DTGS (Deuterated triglycine sulfate) detector. Overall, 32 scans/spectrum were acquired in the 4000–650 cm^−1^ range with a resolution of 4 cm^−1^. Samples were analysed without any previous treatment. To test repeatability, analyses were performed in triplicate and average spectra were used. Spectra were elaborated using the PE Spectrum software version 10.5.1, purchased with the instrument.

### 2.12. Statistical Analysis

All the analyses were performed in triplicate. The data were subjected to analysis of variance (one-way ANOVA test) using SPSS version 19 (SPSS Inc., Chicago, IL, USA) for Windows. The significant level was established at *p* ≤ 0.05 using Duncan’s test.

## 3. Results and Discussion

### 3.1. Proximate Composition

Whole hemp seeds contain approximately 25–35% (by weight) of lipids and 20–25% crude proteins [1,17]. The results of the current study are consistent with the previously established ranges since seeds from *Fedora* cv. exhibited an average content of 27.2 g 100 g^−1^ of lipids and 24.8 g 100 g^−1^ of crude proteins (Table 1).

In regards to flour obtained after oil extraction, a few studies confirmed the expected relative enrichment of carbohydrates and ash, beyond, obviously, a drop of the lipid content [25]. 

Ash represents the total mineral content of a sample, made by inorganic components. The *Fedora* cv. contained an average of 5.3 g 100 g^−1^ (Table 1) ashes, in agreement with Vonapartis et al. [17], who reported ash values ranging from 5.1 to 5.8 g 100 g^−1^ in hemp seeds from ten different *cultivars*.

Total carbohydrates content of *Fedora* cv., including cellulose, hemicellulose, lignin, soluble, and insoluble fibres, was 41.6% and 38.1% (*w/w*) for flour and seed, respectively.

### 3.2. Total Polyphenolic Contents and Antioxidant Activity of Hemp Products

Phenolic compounds primarily contribute to the overall antioxidant capacity of a matrix. They may have important effects on the stability, sensory, and nutritional characteristics of food products, including oil, and may prevent their deterioration through quenching radical reactions responsible for lipid oxidation [26]. 

**Figure 1:**
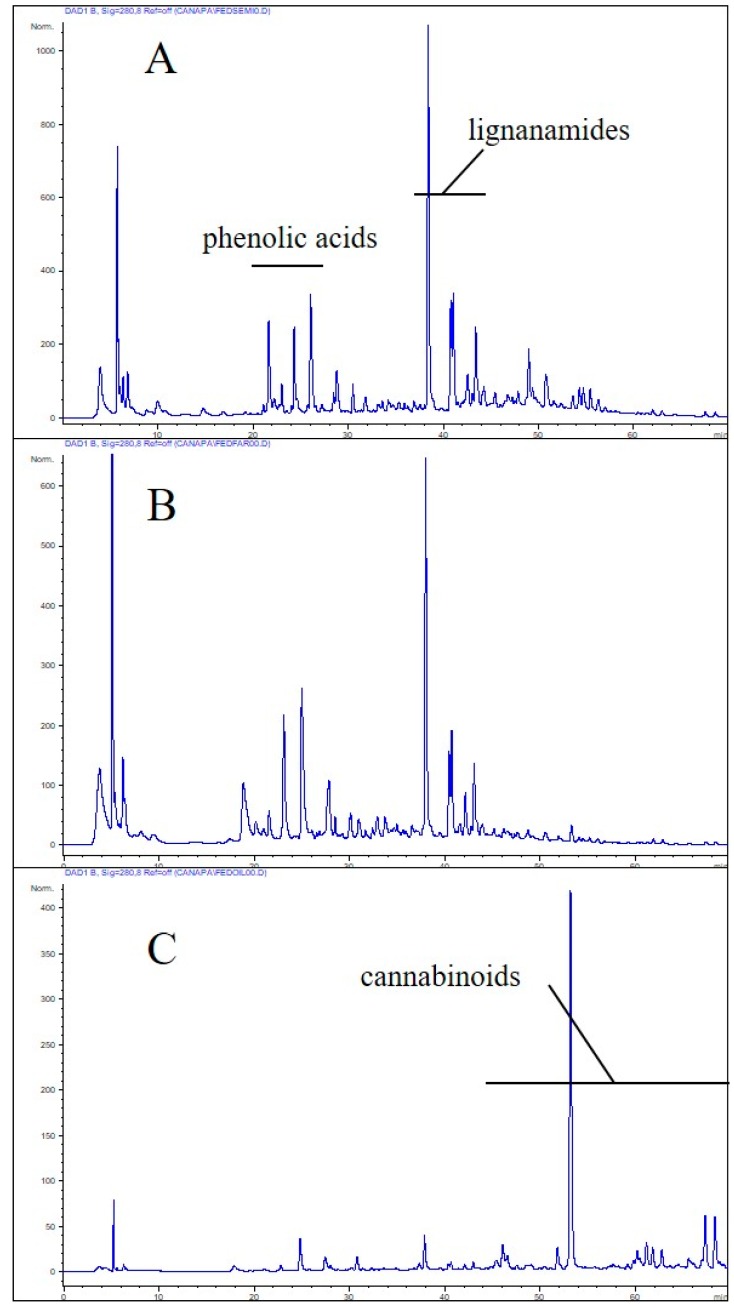
Reversed phase-high performance liquid chromatography (RP-HPLC) chromatograms of hydroalcoholic extracts from seeds (**A**), flour (**B**) and oil (**C**) of hemp *Fedora* cv. The seed and flour extracts share a very similar chromatographic pattern, dominated by lignanamides [27]. As expected, the oil extracts exhibited a different chromatogram, which most likely included simple phenolics and cannabinoid derivatives [28].

Hemp seeds, flour, and oil contained 767 ± 41, 744 ± 29, and 21 ± 5 mg GAE kg^−1^ (gallic acid equivalents) total polyphenols, respectively, determined with the Folin-Ciocalteu (FC) method. Although the level of alcohol-soluble proteins is expected to be very low, FC-based determination of total polyphenols might be biased by several interfering components, including sugars and free amino acids, as well as by the use of unspecific standards. For this reason, polyphenols were semi-quantified also by RP-HPLC adapting the International Olive Council (IOC) method for the determination of the olive oil biophenols [19], using caffeic acid (CA) as the standard. Based on the RP-HPLC semi-quantification, hemp polyphenols were 1540 ± 65, 1078 ± 37, and 23.5 ± 4 mg CAE kg^−1^ (caffeic acid equivalent) for flour, seeds, and oil, respectively. While for oil the determination of total polyphenols yielded very similar values between the two methods, they differed significantly for hemp seeds and flour. The RP-HPLC chromatograms of edible hemp resources monitored at 280 nm are shown in Figure 1. 

Mainly due to the discrepancy among the molecular weight between the standard (i.e., gallic acid) and complex polyphenols, most part of which in hemp seeds and flour are classifiable as lignanamides [27], the FC tends to underestimate the total polyphenol content. In the case of olive oil biophenols, the values obtained with the FC and HPLC-based (the IOC one) methods differ each other by a factor of 2.3–2.6, according to a recent evaluation [20]. For matrices other than the olive oil, this correlation factor should be determined case-by-case. 

Anyhow, our results suggest that edible hemp products may represent a valuable source of phenolic substances, some among them with peculiar structural and functional rendering hemp suitable to design novel nutraceuticals, functional foods or supplements.

**Figure 2:**
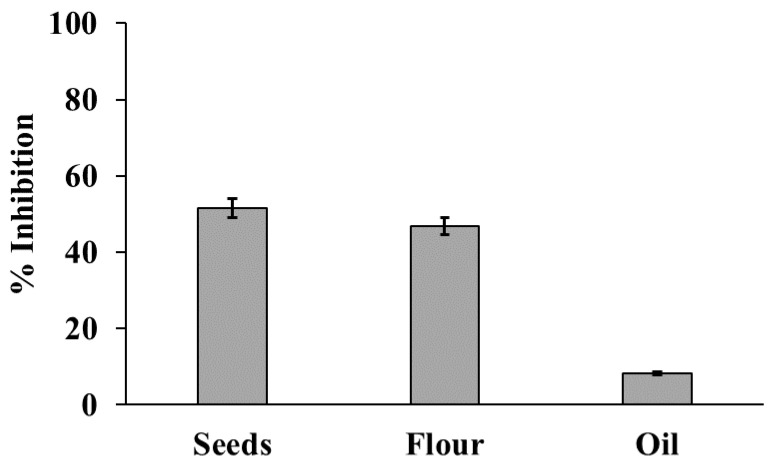
Antioxidant capacity (DPPH test) of seeds, flour, and oil hemp extracts (0.2 g mL^−1^). The results are expressed as inhibition %. Trolox^®^ was used as a positive control for DPPH inhibition (see text). Data are means ± SD of three independent determinations.

The DPPH free radical-scavenging activity of extracts from hemp resources expressed as % inhibition (Figure 2), was consistent with the total polyphenol amount, being comparable between seeds and flour (51.5 and 46.8% inhibition, respectively), while it was much lower for oil extracts (8.2% inhibition). We built a calibration curve of inhibition, using Trolox^®^ as the positive control of the assay. The equation of the calibration curve was y = 8.941x + 4.96, R^2^ = 0.998, where y is % inhibition and x are the Trolox^®^ milliequivalents. Thus, based on the DPPH free radical-scavenging activity, the polyphenol content in terms of Trolox^®^ milliequivalents was 5.2, 4.7, and 0.36 for seeds, flour, and oil extracts, respectively.

### 3.3. Saponificable and Unsaponificable Analysis

**Figure 3:**
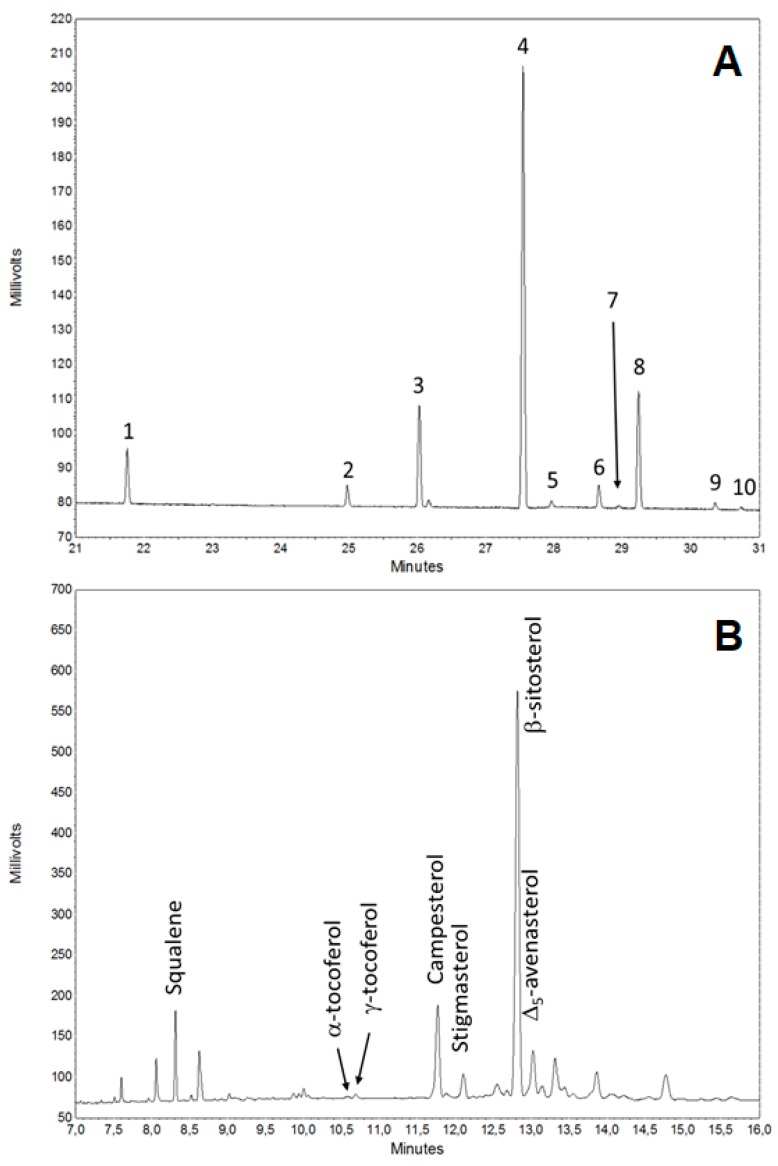
Gas-chromatograms (GC/FID) of hemp (*Fedora* cv) oil fatty acids as fatty acid methyl ester (FAME) (**A**) and of the unsaponifiable fraction (**B**). Fatty acid components are assigned in Table 2.

Glycerolipids of hemp seeds, flour and oil were analysed as fatty acid methyl esters (FAME) by GC/FID (Figure 3A). As expected, the fatty acid composition was similar among the three samples and linoleic acid was dominant in all samples, representing more than 56% of the fatty acids content (Table 2). Other unsaturated fatty acids were linolenic (14.55–15.02%), oleic (12.74–12.79%), and γ-linolenic (2.94–3.03%) acids. Major saturated acids were palmitic (7.03–7.35%) and stearic (2.62–2.78%) acids. 

Our results were in good agreement with the determinations by other authors, such as for instance those reported for cultivars other than Fedora [27,29].

Notably, PUFAs account for about 75% in seeds, oil, and flour, followed by mono-unsaturated (MUFAs; 13%) and saturated fatty acids (SFAs; about 10%).

The ratio of linoleic (ω-6)-to-α-linolenic (ω-3) acids approaches 4:1 indicating a positive nutritional profile of all hemp products, according to a consensus recommendation of several nutritional societies [30]. This property candidate’s hemp oil and derived resources among the food matrices with an optimal PUFA balance, against an average ω-6 to ω-3 ratio of about 10:1 that currently is characterizing the diet of industrialized countries. 

After saponification of glycerolipids, squalene, phytosterols, and tocopherols were determined by GC/FID as well. Among the phytosterols, β-sitosterol, campesterol, and stigmasterol were the most abundant. β-sitosterol is effective to reduce hypercholesterolemia, beyond possessing antiviral, antifungal, and anti-inflammatory properties [31]. Plant sterols reduce plasma cholesterol levels by blocking cholesterol absorption through crystallization and co-precipitation. Within the intestinal lumen, phytosterols reduce cholesterol solubility by excluding it from lipid micelles, thereby preventing its absorption. In addition, sterols compete with cholesterol for intestinal uptake [32].

In agreement with other reports, β-sitosterol, campesterol, Δ_5_-avenasterol, and stigmasterol (in order of decreasing abundance) appeared as the main phytosterols in seeds, oil, and flour products (Table 2). In contrast, brassicasterol was not detected under our experimental workflow.

An exemplificative GC/FID chromatogram of the unsaponificable fraction of hemp oil is shown in Figure 3B, along with the assignment of the main components relying on the comparison of retention times by with pure standards.

The squalene content of oil was about 43.5 mg kg^−1^, which is within the ranges of 20–300 mg kg^−1^ reported for most edible oils [33]. Amount of squalene in seed and flour lipid fractions was comparable to oil (49.3 and 40.1 mg kg^−1^, respectively). Several studies have highlighted the possible beneficial effects associated with the intake of squalene from food.

Among tocopherols, γ-tocopherol was the most abundant isomer with a content higher than 5 mg kg^−1^ of oil, followed by α-tocopherol (about 3 mg kg^−1^).

A series of additional minor components (aliphatic alcohols, hydrocarbon, etc.) were not quantified in the present study.

### 3.4. Minerals Content

The determination of macro- and some trace elements in hemp seeds, flour, and oil is reported in Table 3. Mg and K were the dominant macro-elements in analysed samples, followed by Ca and finally Na, which occurred at a much lower concentration. All macro-elements had a higher concentration in flour than the seeds and, as expected, both were richer in macro-elements than the oil. 

Interestingly, flour was very high in K, suggesting that flour can contribute to regulating heartbeats, maintain fluid balance, and promote in muscle contraction. Flour was high in Mg as well, which supports nerve and muscle functions and helps to maintain the ionic balance integrity of cells.

Ca occurred in significantly high concentration in seeds (944.41 mg kg^−1^) and was more than double in the flour (1907.20 mg kg^−1^). 

Based on previous determinations, these minerals in hemp seeds and flour cover rather wide ranges. The values determined in the current study were below the ones by Mihoc et al. [34] but were in line with Zerihun et al. [35]. This discrepancy may be due to different reasons: (a) A different soil composition; (b) differences in crop annuity; (c) the use of fertilizers based on nitrogen, which can influence the highlighted relationship between increased nitrogen fertilizer and Na reduction in plants [36].

Among the micro-elements (Table 3), Fe showed the highest concentrations followed by Zn, Mn, and Cu, in all the hemp resources, according to previous data [33,37], while Mo, Ni, and Co occurred in minor amounts. 

Fe is very important for human nutrition, because of its role in the formation of haemoglobin. Fe concentrations in the analysed samples fell within the range of values already reported, ranging between 7.8 [38] and 140 mg kg^−1^ [1]. In contrast, we detected lower amounts of Mn and Zn, other important enzyme co-factors, compared to other authors [1,37].

Cu also contributes to redox homeostasis and metabolism. The average concentrations of Cu in whole hemp seeds vary between 7 and 13 mg kg^−1^ [34,35], in line with our determinations. However, Cu accumulation in seeds can vary, depending on its concentration in the soil, rather than on hemp varieties [34]. 

Mo concentration in the oil (0.57 mg kg^−1^) was slightly higher than seeds (0.50 mg kg^−1^) and higher than flour (0.19 mg kg^−1^). Pb is a potent pollutant in the atmosphere with a bioaccumulation effect. In all the analysed samples, Pb was abundantly below 200 mg kg^−1^, which is the safety threshold established by the Regulation EC [39].

Ni is usually present naturally in the soil in small quantities. In all analysed samples, Ni was less abundant than previously reported by others [34,35].

### 3.5. ATR-FTIR Analysis

**Figure 4:**
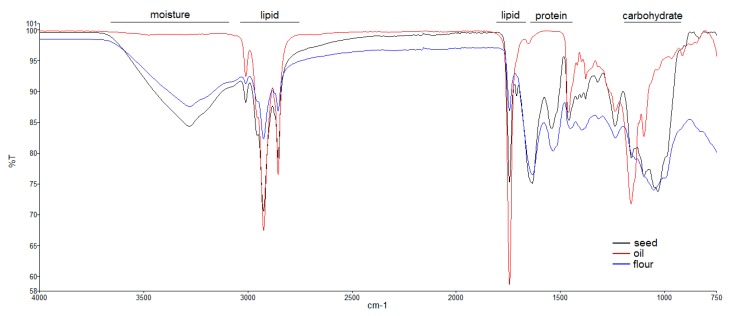
Comparative ATR-FTIR spectroscopy analysis of the three edible hemp products (*Fedora* cv): Seed, oil, and flour. The spectral regions specific to macronutrients (lipids, proteins, and carbohydrates) are indicated in the figure. Protein amines also contribute to the bands in the 3500–3000 cm^−1^ spectral region, indicated as “moisture”.

The ATR-FTIR spectra were used to identify the functional groups of the macronutrients based on the IR absorption in typical spectral regions and Errore di traduzioneto associate characteristic profiles to examined samples. ATR-FTIR spectra for seeds, oil, and flour samples from *Fedora cultivar*, overlaid in Figure 4, clearly evidenced the characteristic bands of macronutrients, including proteins, carbohydrates, lipids, and moisture [40]. All spectra showed the typical carbonyl band at 1744 cm^−1^ and those of hydrogen/carbon bond (alkane, alkene) stretch in the region 2700–3010 cm^−1^, which were obviously dominant in the oil sample and progressively less intense in seeds and flour due to a decreased lipid content (33% and 13%, respectively). 

Only the spectra of seeds and flour presented the broad band centred at 3280 cm^−1^ typical of N-H stretch of secondary amine. Consistently, the protein amide I due to C=O stretching vibrations (1637 cm^−1^) and amide II of the C-N stretching vibrations in combination with N-H bending (1535 cm^−1^) bands only occurred in the spectra of seeds and flour samples. The protein characteristic bands relatively intensified in flour spectra, due to oil depletion. 

Carbohydrates possess strong and characteristic IR absorptions between 1200 and 750 cm^−1^ (fingerprint region) relevant to coupling and combination of stretching/deformation or vibrational modes of individual bonds in the molecular skeleton. Both seeds and flour had intense IR bands assigned to carbohydrates, although the profile in the fingerprint region was significantly different between the two samples. As expected, these bands were missing in the oil. However, such a preliminary suggestion requires opportune validation through dedicate investigations. 

## 4. Conclusions

The results of our study confirm the nutritional features of hemp seed and oil but also highlight the possibility to utilise the waste resources (flour cakes) after oil extraction as a valuable source of natural antioxidants meant for preparing functional food products and nutraceuticals. The edible products, seeds, oil, and flour obtained from a single cultivar had a similar chemical and nutritional composition in terms of acidic and phytosterol profile, phenolic content, antioxidant activity, and content of macro- and micro-elements, even though the relative content of some specific compounds varied in oil, compared to seeds and flour.

ATR-FTIR spectra provide peculiar profiles for the samples, related to the changing balance among nutrients. This technique can be considered as a rapid tool for the chemical gross evaluation of macronutrients of hemp resources to be used in food formulation.

## Figures and Tables

**Table 1:** Proximate composition of flour and seeds of hemp *Fedora* cv.

*Fedora* cv	Moisture (%)	Protein (%)	Lipids (%)	Carbohydrates (%)	Ash (%)
Flour	7.9 ± 0.9	30.7 ± 1.2	13.6 ± 1.9	41.6 ± 2.5	6.2 ± 0.5
Seeds	7.3 ± 0.8	24.8 ± 1.1	24.5 ± 2.0	38.1 ± 2.5	5.3 ± 0.6

**Table 2:** Composition of fatty acids and unsaponifiable components in lipid extracts from hemp edible products (*Fedora* cv).

		Flour Lipids	Cold Pressed Oil	Seed Lipids
N.	Component (Area %)	Fatty Acids		
**1**	Palmitic, C16:0	7.35 ± 0.35	7.15 ± 0.42	7.03 ± 0.31
**2**	Stearic, C18:0	2.62 ± 0.25	2.73 ± 0.21	2.78 ± 0.38
**3**	Oleic, C18:1 ω-9*c*	12.79 ± 1.14	12.75 ± 1.10	12.74 ± 1.25
**4**	Linoleic, C18:2 ω-6*c*	56.42 ± 3.98	56.08 ± 3.05	56.16 ± 3.45
**5**	Arachidic, C20:0	0.74 ± 0.07	0.89 ± 0.05	0.81 ± 0.02
**6**	γ-Linolenic, C18:3 ω-6	3.00 ± 0.40	3.03 ± 0.42	2.94 ± 0.37
**7**	*cis*-11-Eicosenoic, C20:1	0.45 ± 0.05	0.26 ± 0.02	0.37 ± 0.07
**8**	α-Linolenic, C18:3 ω-3	14.55 ± 1.47	14.89 ± 1.18	15.02 ± 1.12
**9**	*cis*-11,14-Eicosadienoic, C20:2	0.82 ± 0.27	1.03 ± 0.32	0.99 ± 0.21
**10**	Behenic, C22:0	0.29 ± 0.05	0.20 ± 0.06	0.27 ± 0.03
Σ-SFA		11.00 ± 0.42	10.97 ± 0.47	10.89 ± 0.49
Σ-MUFA		13.24 ± 1.14	13.01 ± 1.10	13.11 ± 1.25
Σ-PUFA		74.79 ± 4.27	75.03 ± 3.31	75.11 ± 3.65
Σ-PUFA/Σ-SFA		6.80	6.84	6.90
**Component (mg kg^−1^)**		**Unsaponificable Fraction**		
Squalene		40.1 ± 6.1	43.5 ± 5.2	49.3 ± 5.6
α-tocoferol		3.2 ± 0.7	2.7 ± 0.5	3.5 ± 0.9
γ-tocoferol		5.8 ± 0.6	5.0 ± 0.8	5.5 ± 1.3
Brassicasterol		-	-	-
Campesterol		125.1 ± 8.2	117.4 ± 9.3	115.4 ± 10.3
Stigmasterol		27.4 ± 2.8	28.2 ± 2.1	24.7 ± 2.5
β-sitosterol		528.4 ± 28.8	530.4 ± 25.4	536.1 ± 31.5
Δ_5_-avenasterol		69.6 ± 8.6	72.6 ± 6.6	76.2 ± 8.4

**Table 3:** Composition of macro and microelements of seed, flour, and oil of *Fedora* cv.

(mg kg^−1^)	Seed	Flour	Oil	(µg kg^−1^)	Seed	Flour	Oil
Na	67.54	90.55	88.79	Fe	97.97	152.47	1.71
Ca	944.41	1907.20	53.86	Cu	5.02	11.94	1.48
Mg	2682.13	2310.54	199.07	Zn	48.43	54.68	0.91
K	2517.35	5064.45	20.72	Mn	44.42	94.71	0.82
				Ba	2.04	4.08	0.43
				Mo	0.50	0.19	0.57
				Co	0.03	0.03	0.06
				Al	2.12	3.48	1.83
				Ni	0.79	0.85	0.03
				Pb	0.04	0.03	0.09
